# Characterisation of light responses in the retina of mice lacking principle components of rod, cone and melanopsin phototransduction signalling pathways

**DOI:** 10.1038/srep28086

**Published:** 2016-06-15

**Authors:** Steven Hughes, Jessica Rodgers, Doron Hickey, Russell G. Foster, Stuart N. Peirson, Mark W. Hankins

**Affiliations:** 1 The Nuffield Laboratory of Ophthalmology, Sleep and Circadian Neuroscience Institute, Nuffield Department of Clinical Neurosciences, University of Oxford, Sir William Dunn School of Pathology, OMPI G, South Parks Road, Oxford, OX1 3RE, UK

## Abstract

*Gnat*^*−/−*^, *Cnga3*^*−/−*^, *Opn4*^*−/−*^ triple knockout (TKO) mice lack essential components of phototransduction signalling pathways present in rods, cones and photosensitive retinal ganglion cells (pRGCs), and are therefore expected to lack all sensitivity to light. However, a number of studies have shown that light responses persist in these mice. In this study we use multielectrode array (MEA) recordings and light-induced *c-fos* expression to further characterise the light responses of the TKO retina. Small, but robust electroretinogram type responses are routinely detected during MEA recordings, with properties consistent with rod driven responses. Furthermore, a distinctive pattern of light-induced *c-fos* expression is evident in the TKO retina, with *c-fos* expression largely restricted to a small subset of amacrine cells that express disabled-1 (Dab1) but lack expression of glycine transporter-1 (GlyT-1). Collectively these data are consistent with the persistence of a novel light sensing pathway in the TKO retina that originates in rod photoreceptors, potentially a rare subset of rods with distinct functional properties, and which is propagated to an atypical subtype of AII amacrine cells. Furthermore, the minimal responses observed following UV light stimulation suggest only a limited role for the non-visual opsin OPN5 in driving excitatory light responses within the mouse retina.

Light detection in the retina is mediated by three distinct classes of photoreceptor; rods, cones and melanopsin expressing photosensitive retinal ganglion cells (pRGCs)^1^,^2^. These different classes of photoreceptors perform different physiological roles and are characteristically sensitive to different wavelengths of light^2^ (rhodopsin λ_max_ 498 nm; MWS cone opsin λ_max_ 508 nm; UVS cone opsin λ_max_ 360 nm, melanopsin λ_max_ 479 nm^3^). *Gnat*^*−/−*^, *Cnga3*^*−/−*^, *Opn4*^*−/−*^ triple knockout (TKO) mice^4^ lack essential components of the phototransduction pathway present in each of these classes of photoreceptor (rod α transducin within rods, cone-specific cyclic nucleotide gated channel α3 subunit within cones, and melanopsin within pRGCs) and are expected to lack all sensitivity to light (for discussion see^5^). However, although highly attenuated, several studies have shown that these mice do retain a residual perception of light. A small but significant pupil constriction is observed in response to bright light stimuli^4^, and these mice also show some level of light avoidance^6^. Furthermore, small flash electroretinogram (ERG) responses are evident in these mice^5^. Light responses have also been recorded from the lateral geniculate nucleus (LGN) and light-induced expression of *c-fos* (a marker of neuronal activation) is evident in the visual cortex of these mice^5^. However, the cellular mechanisms underlying these residual light responses and the retinal pathways by which they are propagated remain to be determined. There is evidence that transducin independent signalling pathways may exist within rod photoreceptors^7^,^8^, and light responses have been recorded from a small subset of rod photoreceptors in *Gnat*^*−/−*^ mice^9^. Studies by Allen *et al*.^5^ indicate that rods are responsible for driving ERG responses in TKO mice, yet this response is likely mediated by low levels of cone transducin (Gnat2) expressed within rod photoreceptors^5^. Thus it would seem that multiple novel light signalling pathways may be present within rod photoreceptors. It is also possible that a number of other non-visual opsins may contribute to light responses observed in the TKO (and wild type) retina. One intriguing candidate is OPN5, a UV-sensitive (λ_max_ 380 nm) Gi-coupled non-visual opsin^10^,^11^ which is expressed within a subset of retinal ganglion cells of the mouse retina^11^,^12^,^13^, and is reported to drive entrainment of retinal circadian oscillators to light dark cycles independent of rods, cones and melanopsin^13^.

Here we use multiple electrode array (MEA) recordings, and light-induced expression of the immediate early response gene *c-fos* to further characterise the light responses of the TKO retina. Using different wavelengths of light to preferentially stimulate different classes of retinal photoreceptors, our data confirm that light responses do indeed persist within the retina of TKO mice, and are consistent with the survival of a novel rod based signalling pathway that includes the downstream activation of a population of atypical AII amacrine cells. Furthermore our results suggest that expression of the UV-sensitive non-visual opsin OPN5 is not sufficient to drive excitatory light responses in the retina of TKO mice.

## Results

### Multiple electrode array recordings of light responses in the TKO retina

**Figure 1:**
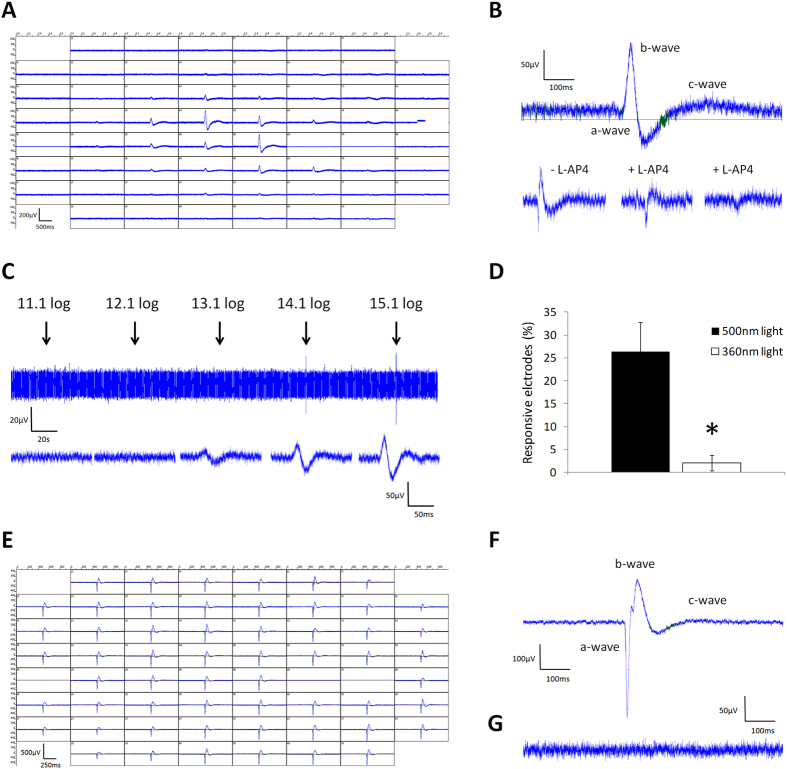
Multiple electrode array (MEA) recording of light response from TKO retina explants. (**A**) Image showing micro ERG type responses recorded from the TKO retina following stimulation with 500 nm light (500 ms, 15.1 log photons/cm^2^/s). Panels show the responses recorded from all recording electrodes of a single MEA chamber. (**B**) An example of a micro ERG response from a single electrode showing the principle components of the ERG response (500 nm light, 500 ms, 15.1 log photons/cm^2^/s). Lower panels show the effect of 100 μm L-AP4 at reducing the *b*-wave component, leading to the isolation of the *a*-wave component (middle), which is rapidly bleached (right). (**C**) Recording from a single electrode showing the amplitude of micro ERG responses elicited by increasing intensities of 500 nm light (500 ms, 11.1 to 15.1 log photons/cm^2^/s). Lower panels show individual responses at higher resolution. (**D**) Graph showing the percentage of electrodes exhibiting micro ERG type light responses following 500 nm light pulses (500 ms, 15.1 log photons/cm^2^/s) and 360 nm light pulses (500 ms, 14.9 log photons/cm^2^/s). * Indicates p < 0.01. (**E**) Image showing micro ERG type responses recorded from the normal wildtype retina following stimulation with 500 nm light (500 ms, 15.1 log photons/cm^2^/s). (**F**) Image showing an example of micro ERG responses recorded from a single electrode in wildtype retina (500 nm light, 500 ms, 15.1 log photons/cm^2^/s). Note the increased amplitude of *a*-waves compared to TKO responses. (**G**) Image showing an example of micro ERG responses recorded from a single electrode in degenerate *rd/rd cl* retina lacking rods and cones (500 nm light, 500 ms, 15.1 log photons/cm^2^/s). Note the lack of micro ERG type responses in the *rd/rd cl* retina.

Electroretinogram (ERG) type responses (micro ERGs) can be recorded from retinal explants using multiple electrode arrays (MEAs), and can be used to measure the electrical activity of different cell types in the retina^14^. MEA recordings from the retina of TKO mice showed small localised, robust ERG type responses following stimulation with 500 nm light (500 ms duration, 15.1 log photons/cm^2^/s) (Fig. 1A). The specific components of the ERG waveform could be distinguished with prominent *b*-waves (driven by ON bipolar responses) and *c*-waves (driven by photoreceptors and pigment epithelium), and small *a*-waves (driven by rod and cone photoreceptors) that were typically more difficult to detect (Fig. 1B). Application of 100 μM L-AP4 (a group III metabotropic glutamate receptor agonist) to block bipolar cell responses and reduce *b*-wave amplitude facilitated the isolation of *a*-waves, although these remained relatively small in nature and were easily bleached (Fig. 1B). In total, 132 of 279 electrodes from n = 5 retina showed micro ERG type responses following stimulation with 500 nm light (Fig. 1D). These responses showed a dose dependent effect of light with increasing amplitude of responses detected on individual electrodes following increasing intensity of light (Fig. 1C). Repeated application of moderate intensity 500 nm light (500 ms, 14.1 log photons/cm^2^/s) typically resulted in reproducible responses to light pulses spaced 60 s apart, yet stimulation with the brightest 500 nm light stimuli used (500 ms, 15.1 log photons/cm^2^/s) resulted in relatively rapid bleaching and reduction of subsequent responses (full data not shown). Compared to 500 nm stimulation, responses to 360 nm UV light were significantly reduced with micro ERG type events detected on only 8 of 222 electrodes from n = 4 retina, t-test p = 0.01 (Fig. 1D), and only detected at the highest intensity of UV light used (500 ms, 14.9 log photons/cm^2^/s). In all cases where responses were observed to UV light, these responses were smaller in amplitude compared to responses recorded from the same electrode following 500 nm light stimuli of similar intensity (Supplementary Figure 1A). This effect was observed irrespective of the order in which 500 nm and 360 nm light flashes were applied, and responses to UV light were minimal even when applied as the first light stimuli to fully dark-adapted retinae (full data not shown).

The properties of ERG events recorded from TKO retina showed a number of clear differences compared to those recorded from wild type (WT) retina (Fig. 1E,F and Supplementary Figure 2). In the WT retina ERGs were typically much larger in amplitude, with dark-adapted *a*-wave amplitudes typically ranging from 400–1000 μV, and were consistently detected on all electrodes at light onset (ON responses) (100% of electrodes from n = 6 retina) (Fig. 1E,F). Additionally, unlike the TKO retina, in the WT retina ERG type responses were observed at light offset (OFF responses) for a high percentage of electrodes (typically 30–60%) (Supplementary Figure 2C). As expected, ERG type responses were completely absent from the retina of degenerate *rd/rd* mice lacking rod and cone photoreceptors (0 responsive electrodes recorded from >10 retina) (Fig. 1G and Supplementary Figure 2E).

In addition to micro ERG type events, we also investigated changes in spike firing rate consistent with the activation of ON, OFF or ON-OFF retinal ganglion cells. However, we failed to detect any changes in spike firing rate of ganglion cells following 500 nm light stimulation of TKO retina explants (0 of 279 electrodes from n = 5 retina, stimulus ranging from 11.1–15.1 log photons/cm^2^/s, and from 500 ms to 30 seconds in duration). By contrast, a single electrode did show a notable change in spike firing rate following stimulation with 360 nm UV light (1 of 222 electrodes, from n = 4 retina, responding to a 1 s pulse of 360 nm light at 14.9 log photons/cm^2^/s) (Supplementary Figure 1B–D). For this electrode, the increase in spike firing rate coincided precisely with the onset of stimulation and showed a sluggish light response, with spike firing rate increasing over time and reaching maximal levels ~15 seconds after light onset. The activity of this electrode showed only limited signs of recovery with spike firing remaining elevated for 20–30 minutes following stimulation (full data not shown). By comparison, transient changes in spike firing rate were consistently recorded from wild type retinae stimulated under similar conditions, consistent with rod/cone and melanopsin driven light responses respectively (Supplementary Figure 2A–D). Again, consistent with the complete lack of functional rod and cone photoreceptors, only melanopsin type light responses were detected from the degenerate *rd/rd cl* retina, with rod and cone driven responses (including ERGs) completely absent (Supplementary Figure 2E–G).

### *c-fos* as a marker of cellular light responses in the TKO retina

**Figure 2:**
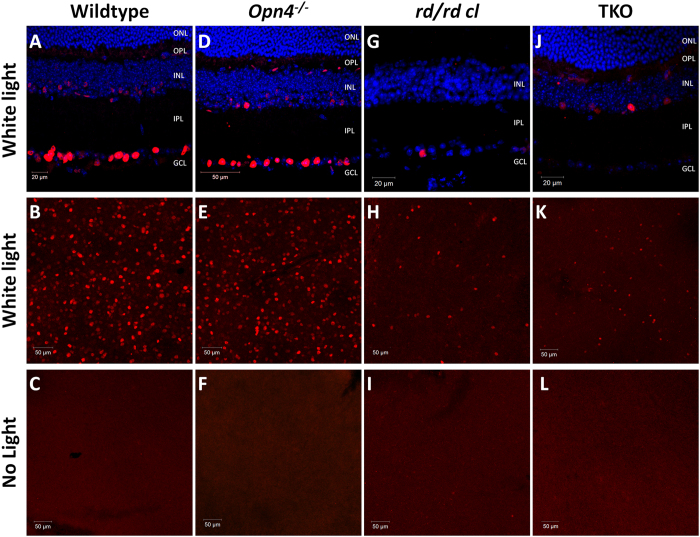
Light-induced expression of *c-fos* expression in the wild type, Opn4^*−/−*^*, rd/rd cl*, and TKO retina. Images from retinal sections (**A**,**D**,**G**,**J**) and whole-retin**a** flat-mounts (**B**,**E**,**H**,**K**) showing the loc**a**lisation and distribution of *c-fos* expression observed in wild type retina (**A,B**), *Opn4*^*−/−*^retina (**D,E**), degenerate *rd/rd cl* retina (**G,H**) and the TKO retina (**J,K**) following white light pulses (14.7 log photons/cm^2^/s for 30 mins at ZT16). (**C,F,I,L**) Images of flatmount retina showing levels of *c-fos* expression detected in mice of each genotype following sham light pulses. Flatmount images are generated from merging confocal slices (1 μm in z-axis) collected from the ganglion cell layer to the inner nuclear layer. DAPI nuclear counter stain is shown in blue. White light stimuli lacking any UV component (14.7 log photons/cm^2^/s) and 360 nm UV light stimuli (12.7 log photons/cm^2^/s) were produced by LED light sources.

We next sought to determine the nature of cellular light responses in the TKO retina using expression of the early immediate response gene *c-fos* as a marker of light induced activation *in vivo*^15^,^16^,^17^,^18^,^19^. This approach has the advantage of maintaining normal rod and cone function during *in vivo* light stimulation, whilst also allowing detection of light response in cell types that do not display measurable electrical responses (amacrine cell activation for example). Analysis of *c-fos* expression again shows that cellular light responses persist in the retina of TKO mice. These responses are largely attenuated compared to those observed in wild type and *Opn4*^*−/−*^ retinae, and show a distinctively different pattern of expression compared to the *rd/rd cl* retina where *c-fos* expression is largely restricted to pRGCs^15^,^19^ (Fig. 2 and Supplementary Figures 4 and 5).

**Figure 3:**
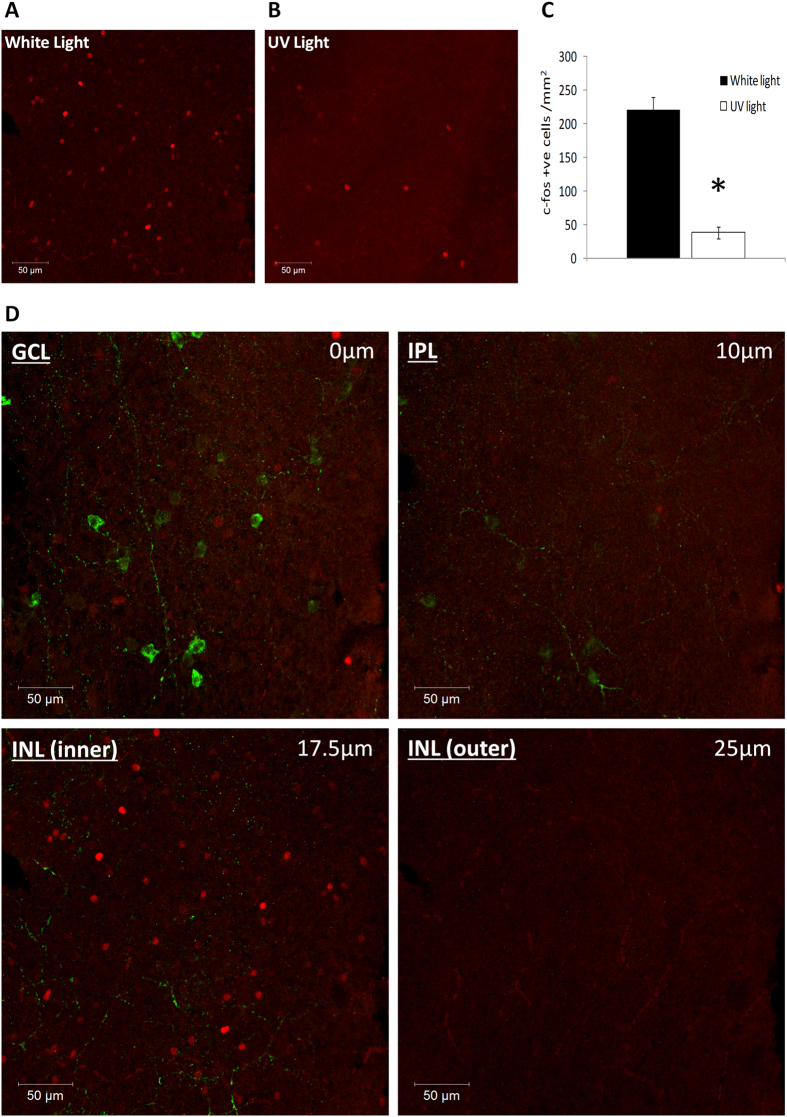
Light-induced *c-fos* expression in the TKO retina following white light and UV light stimuli. (**A,B**) Levels of light-induced *c-fos* observed in the TKO retina following white light (14.7 log photons/cm^2^/s for 30 mins at ZT16) and UV light pulses (12.7 log photons/cm^2^/s). (**C**) Graph showing the density of *c-fos* positive cells detected in the TKO retina following white light and UV light pulses. * Indicates p = 7.3E-8. (**D**) Individual confocal slices (2.5 μm in z-axis) showing the distribution of *c-fos* positive cells (*red*) in the ganglion cell layer (GCL) and inner nuclear layer (INL) of the TKO retina. Labelling of M1-type pRGCs (green) (via β-gal labelling of tauLacZ reporter incorporated into TKO mice) that project to the INL is shown to confirm the location of cells on the innermost surface of the INL. Location of the ganglion cell layer (GCL), inner plexiform layer (IPL) and inner nuclear layer (INL), as well as the depth from the GCL are indicated by the relevant labels.

Unlike the wild type retina, where the highest levels of *c-fos* expression are detected in the ganglion cell layer, in the TKO retina light-induced *c-fos* expression (white light LED, 30 mins, 14.7 log photons/cm^2^/s) is largely confined to a rare subset of cells located on the inner surface of the inner nuclear layer (INL) (94.9%; 468 of 492 *c-fos* positive cells counted from n = 3 retina) with a smaller number of responsive cells also detected in the ganglion cell layer (GCL) (5.1%) (Fig. 3D). Detectable levels of *c-fos* expression were almost completely absent in TKO mice (and wild type, *Opn4*^*−/−*^, and *rd/rd cl* retina) not receiving light pulses (Fig. 2C,F,I,L). Based on analysis of whole retina flatmounts the density of *c-fos* positive cells in the TKO retina was 221.0 ± 19.1 cells/mm^2^ (counts performed on n = 10 0.22 mm^2^ images collected from n = 3 retina), with no obvious gradient in distribution of these cells observed across the retina (Fig. 3). The number of *c-fos* positive cells detected in the TKO retina was significantly reduced following stimulation with UV light (360 nm LED, 30 mins, 12.7 log photons/cm^2^/s) compared to white light (38.6 ± 8.5 and 221.0 ± 19.1 cells/mm^2^ respectively, p = 7.3E-8) (Fig. 3A–C and Supplementary Figure 3), with the majority of responsive cells again located in the INL (92.5%; 80 of 86 *c-fos* positive cells counted from n = 3 retina).

### Induction of *c-fos* is restricted to distinct subsets of amacrine cells in the TKO retina

**Figure 4:**
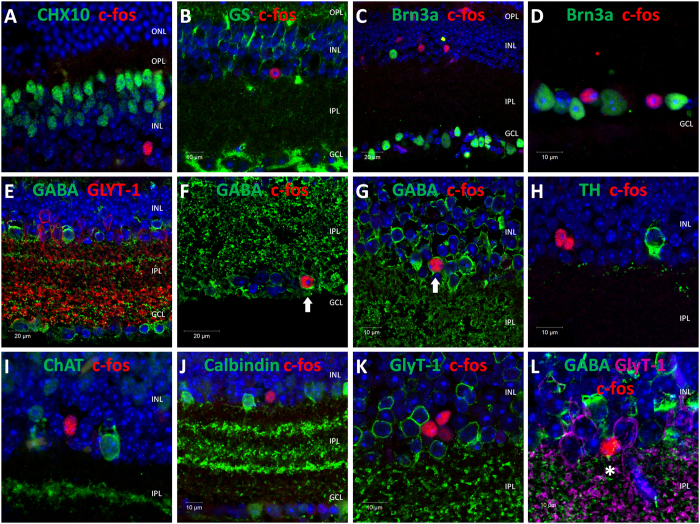
Identification of *c-fos* positive cells in the TKO retina. Images showing co-localisation of *c-fos* with specific retina cell types in the TKO retina following white light pulses (14.7 log photons/cm^2^/s for 30 mins at ZT16), 30 mins, ZT16). (**A**) *c-fos* and CHX10 (bipolar cell marker). (**B**) *c-fos* and glutamine synthetase (Müller cell marker). (**C,D**) *c-fos* and Brn3a (RGC cell marker). (**E**) GABA and GlyT-1 (GABAergic and glycinergic amacrine cells). (**F,G**) *c-fos* and GABA (GABAergic amacrine cell marker). (**H**) *c-fos* and tyrosine hydroxylase (TH) (dopaminergic amacrine cells). (**I**) *c-fos* and ChAT (starburst amacrine cells). (**J**) *c-fos* and calbindin (marker of multiple cell types including amacrine and horizontal cells). (**K**) *c-fos* and GlyT-1 (glycinergic amacrine cells). (**L**) *c-fos* and GABA and GlyT-1 (GABAergic and glycinergic amacrine cells). DAPI nuclear counterstain is shown in blue. Arrows indicate *c-fos* positive GABA positive amacrine cells. Asterix indicates a *c-fos* positive cell that is negative for both GABA and GlyT-1. ONL, outer nuclear layer; OPL, outer plexiform layer; INL, inner nuclear layer; IPL, inner plexiform layer; GCL, ganglion cell layer. For full description of antibodies see Table 1.

Following the identification of light activated cells in the retinae of TKO mice, we sought to determine the identity of these cells using a range of well characterised antibody markers of specific retinal cell types. Based on the location of these cells at the inner surface of the INL, and to a lesser extent the GCL, it is likely that these cells represent some type of amacrine cell and or displaced retinal ganglion cell (RGC). In keeping with this assumption, detectable levels of *c-fos* expression were absent from CHX10 positive bipolar cells (Fig. 4A), and glutamine synthetase positive Müller cells (Fig. 4B). *c-fos* expression was also absent from Brn3a positive retinal ganglion cells (RGCs), including RGCs located in the GCL and also displaced RGCs located within the INL (Fig. 4C,D). Detectable levels of *c-fos* expression were also absent from M1-type melanopsin expressing photosensitive retinal ganglion cells (pRGCs) (Fig. 3D) (lacking expression of melanopsin in the TKO retina and identified based on expression of a β-gal reporter expressed selectively within M1-type pRGCs^20^,^21^).

The vast majority of amacrine cells in the mouse retina are either glycinergic or GABAergic (Fig. 4E)^23^. Double labelling with *c-fos* and GABA antibodies indicates that approximately 15% of *c-fos* cells identified in the TKO retina are GABA-positive amacrine cells (Fig. 4F,G). These GABA positive amacrine cells accounted for almost all of the *c-fos* positive cells detected in the GCL (>95%) (Fig. 4F), but represented only a small percentage of the *c-fos* positive cells identified in the INL (~10%) (Fig. 4G). However, it was not possible to determine the specific identity of these GABAergic amacrine cells using the antibodies tested in this study. Detectable levels of *c-fos* expression were absent from tyrosine hydroxylase (TH) positive dopaminergic amacrine cells (Fig. 4H), choline acetyltransferase (ChAT) positive starburst amacrine cells (Fig. 4I) and calbindin positive amacrine cells (Fig. 4J), all of which are GABAergic and show light-induced *c-fos* expression in the wild type retina (Supplementary Figure 4).

**Figure 5:**
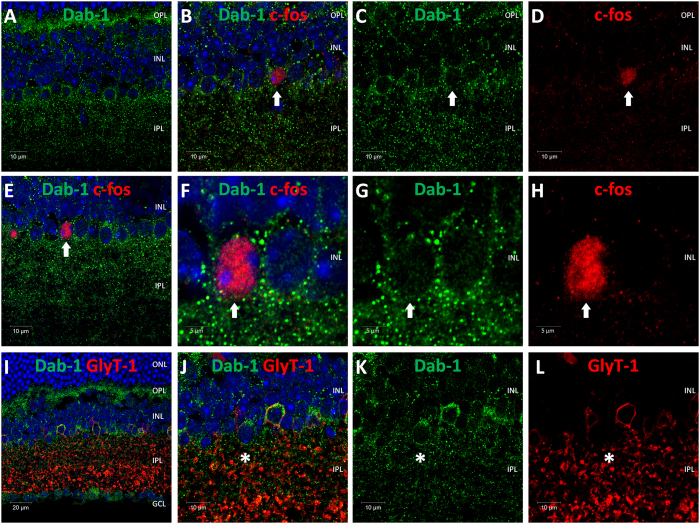
*c-fos* positive cells in TKO retina are Dab1 positive, GlyT-1 negative amacrine cells. Images showing expression of *c-fos* within Dab1 positive, GlyT-1 negative amacrine cells in the TKO retina following white light pulses (14.7 log photons/cm^2^/s for 30 mins at ZT16). (**A**) Disabled-1 (Dab1) antibody labels a high number of cells on the inner surface consistent with labelling of AII amacrine cells. (**B–D**) Series of image panels showing expression of *c-fos* in a small subset of Dab1 positive amacrine cells. (**E**,**F,H**) Further examples showing expression of *c-fos* in a small subset of Dab1 positive amacrine cells. (**I**) Image showing high levels of co-expression of Dab1 and Glycine Transporter-1 (GlyT-1) in AII amacrine cells. (**J–L**) Series of image panels showing a small subset of Dab1 positive AII amacrine cells lack detectable expression of GlyT-1. DAPI nuclear counterstain is shown in blue. Arrows indicate *c-fos* positive Dab1 positive amacrine cells. Asterix indicates a Dab1 positive cell that is negative for GlyT-1. ONL, outer nuclear layer; OPL, outer plexiform layer; INL, inner nuclear layer; IPL, inner plexiform layer; GCL, ganglion cell layer.

Given that the majority of *c-fos* positive cells identified in the INL were not GABA positive, we assumed that they would be glycinergic amacrine cells. However, none of the *c-fos* positive cells detected in the TKO retina were positively stained for glycine transporter-1 (GlyT-1) (Fig. 4K), a classic marker of glycinergic amacrine cells^24^. Triple labelling with GlyT-1, GABA and *c-fos* antibodies confirmed that the majority of *c-fos* positive cells observed in the TKO retina lack expression of either GABA or GlyT-1 (Fig. 4L). Surprisingly, despite lacking expression of GlyT-1 the majority (~70%) of the *c-fos* positive cells identified in the INL of the TKO retina were positively labelled for Disabled-1 (Dab1) (Fig. 5A–H), a marker of Type AII amacrine cells^25^; cells that typically express GlyT-1^26^. Overall, these *c-fos* positive cells represented only a small percentage of the total population of Dab1 positive cells (<5%). As expected from previous studies^25^, double labelling with GlyT-1 and Dab1 antibodies showed a high level of co-expression, with the majority of Dab1 positive cells also labelled for GlyT-1 (Fig. 5I). However, interestingly we did detect a small number of Dab1 positive cells that did not show detectable levels of GlyT-1 expression (<5% of all Dab1 positive cells) (Fig. 5J–L). The location and morphology of these GlyT-1 negative Dab1 positive cells is consistent with the properties of the *c-fos* positive Dab1 positive cells we have identified.

Based on our data, the majority of *c-fos* positive cells identified in the light-pulsed TKO retina are Dab1 positive GlyT-1 negative amacrine cells located at the inner surface of the INL, which seem to represent a distinct subset of Type AII amacrine cells. A smaller number of unidentified GABAergic amacrine cells that were typically located in the ganglion cell layer were also *c-fos* positive. The confinement of *c-fos* expression to these cell types is in contrast to the wild type and *Opn4*^*−/−*^ retina where multiple cell types show light-induced *c-fos* expression, including retinal ganglion cells and multiple subtypes of amacrine cell (Supplementary Figure 4) (see also^27^,^28^), and also the *rd/rd cl* retina where *c-fos* expression is observed predominantly for pRGCs and a subset of dopaminergic amacrine cells (Supplementary Figure 5) (see also^15^,^19^,^29^).

## Discussion

In this study we have used multiple electrode array (MEA) recordings and light-induced expression of *c-fos* to show that cellular light responses persist in the retina of *Gnat*^*−/−*^, *Cnga3*^*−/−*^, *Opn4*^*−/−*^ triple knockout (TKO) mice. MEA recordings from the retinae of TKO mice showed localised but robust micro ERG type responses (including *a-*waves and *b-*waves) following stimulation with 500 nm light, and to a lesser extent 360 nm light. ERG responses recorded from the TKO retina are highly attenuated compared to wild type retinae, but these responses are completely absent from the degenerate *rd/rd cl* retinae where rod and cone photoreceptors are absent. Analysis of light-induced *c-fos* expression again confirms the presence of residual light signalling pathways in the TKO retina. Double labelling experiments indicate that at least two distinct cell types (possibly more) show robust light-induced *c-fos* expression in the TKO retina, although the total number of responsive cells is low. The majority of responsive cells were located on the inner surface of the inner nuclear layer (INL), and are identified as a rare and atypical subset of Type AII amacrine cells that express Disabled-1 (Dab1), but lack detectable expression of the glycine transporter GlyT-1 and GABA. The remaining cells include a small subset of as yet unidentified GABA positive amacrine cells located in both the INL and GCL.

As the function of rods, cones and pRGCs are expected to be eliminated in the TKO retina the obvious question is what pathway is mediating this residual photosensitivity. Given that the loss of melanopsin photopigment completely eliminates endogenous photoresponses from pRGCs^4^,^30^, and that we did not detect melanopsin type responses during MEA recordings from TKO retina it seems highly unlikely that pRGCs are the source of the light responses observed in TKO mice. However, both rods and cones retain expression of their respective photopigments in the TKO retina, where instead a loss of photoreceptor function is induced by removal of an essential component of their respective phototransduction cascades (Gnat1, rod α transducin within rods; Cnga3, cone-specific cyclic nucleotide gated channel α3 subunit within cones). This does therefore leave the possibility that residual phototransduction signalling pathways may remain in either rods or cones, potentially acting via secondary signalling pathways or following some type of functional substitution.

Previous studies have shown that the light driven ERG responses observed in the TKO have spectral sensitivities consistent with the involvement of rod opsin but not cone opsins or melanopsin, with these rod driven responses mediated by low levels of cone transducin (Gnat2) expression in rods^5^. Collectively, our data are consistent with the findings of Allen *et al*., and support a role for rod photoreceptors in driving micro-ERG type events observed during MEA recordings and also light induced expression of *c-fos* in the TKO retina. A role for either rods or cones is supported by the observation that both micro ERG type events and a similar pattern of *c-fos* responsive cells are absent from the retinae of degenerate *rd/rd cl* mice where rod and cone photoreceptors are completely absent. The increased sensitivity of MEA and *c-fos* responses to 500 nm or white light compared to 360 nm UV light would again seem to support a role for rods and not cones. Both cone types of the mouse retina, M-cones and S-cones, express UVS cone opsin and exhibit robust responses to UV light^31^,^32^,^33^, and our previous studies have shown that cones drive robust levels of *c-fos* expression in the mouse retina under similar levels of UV light^15^. The presence of *c*-waves, the rapid and irreversible bleaching observed to bright light stimulation and the lack of OFF responses (a result of cone OFF bipolar cell activity) all indicate rod driven, and not cone driven responses during MEA recordings. Furthermore, given that rods are the principle pathway influencing Type AII amacrine cells^34^,^35^, our *c-fos* data would also seem to support the presence of residual rod based light responses in the TKO retina.

Furthermore, our data indicate only a minimal, if any, role for OPN5 in driving excitatory light responses in the TKO retina. OPN5 is a Gi-coupled UV-sensitive non-visual opsin^10^,^11^ that is expressed in retinal ganglion cells of the mouse retina^11^,^12^, and acts to entrain retinal circadian oscillators to light dark cycles^13^. However, during MEA recordings, we did not detect changes in spike firing rate following stimulation with either 500 nm or 360 nm light (or any other wavelength tested), as would be expected from the activation of retinal ganglion cells. The exception to this was a single electrode that did show a slow sluggish response to 360 nm UV light (but did not respond to previous exposure to 500 nm light) (1 of 222 electrodes), and showed only limited signs of recovery over 20–30 minutes following stimulation. Due to the prolonged nature and the low incidence of such responses, it is unclear whether this represents a genuine ‘photoreceptor mediated’ response to light or a non-specific effect of UV light stimulation in this *in vitro* retina preparation. Given the number of OPN5 expressing RGCs reported in the mouse retina^11^,^12^,^13^ we may have expected more widespread responses should this photopigment be responsible for driving excitatory light responses under these conditions. In addition, the relative lack of *c-fos* expression within the ganglion cell layer of the TKO retina following UV light stimulation again indicates only a minimal, if any, role for OPN5 in driving light induced cellular depolarisation of RGCs in the mouse retina. These findings are in agreement with previous suggestions that OPN5 derived light signals that entrain circadian oscillators within the mouse retina may be non-electrical in nature^13^. However, it is worth noting that OPN5 has been reported to drive excitatory light responses in cells of the quail brain^36^, but see also^37^.

Are the light responses observed in the TKO retina driven by a specialised subset of rod photoreceptors? The mouse retina is rod dominated, with rods representing 97% of all outer retinal photoreceptors. Given that rods seem to retain some level of photosensitivity in the TKO retina, it is not clear why so few cells show signs of *c-fos* activation, or why this expression should be restricted to such a defined sub population of retinal cell types. In the wild-type retina the highest levels of light-induced *c-fos* expression are typically observed for amacrine cells, with lower levels of expression observed for retinal ganglion cells (Supplementary Figure 3). This observation may explain why we detect *c-fos* only in amacrine cells of the TKO retina, as the residual rod based light responses in these mice are too insensitive to drive *c-fos* expression in anything except the most sensitive of cell types (with respect to *c-fos* induction). However, this observation does not explain why so few cells show *c-fos* expression, or why *c-fos* expression is restricted to such a small and distinct subset of cells in the TKO retina. It is possible that the patterning of responsive cells we observe represents a novel retinal signalling pathway, potentially involving only a small subset of specialised rod photoreceptors that in turn couple to a rare and atypical subset of Type AII amacrine cells that are neither glycinergic nor GABAergic (a property described for other subtypes of amacrine cells^38^). It should also be noted that, based on the presence of *b*-waves in ERG type responses reported by Allen *et al*.^5^, and also in MEA recordings presented here, it is clear that rod driven signals in the TKO retina are also propagated to ON bipolar cells. However, even in the wildtype retina, bipolar cells do not typically show high levels of light induced *c-fos* expression under these conditions (Supplementary Figure 3), and thus it is not possible to visualise all components of this novel signalling network using the *c-fos* approach. In support of a specialised subset of rod photoreceptors, previous studies have reported that light responses can be recorded from a small number of rods (less than 1%) within the *Gnat1*^*−/−*^ retina^9^. Heterogeneity in rod responses from *Gnat1*^*−/−*^ retina have also been reported by Woodruff *et al*., with a small percentage of rods showing light responses with significantly higher amplitudes compared to other Gnat1 deficient rods^8^. The apparent rarity of rod driven responses in the *Gnat1*^*−/−*^ retina may offer a potential explanation to why we fail to observe significant changes in retinal ganglion cell firing in the TKO retina despite clear evidence of propagation of signals from rods to bipolar cells, and evidence from Allen *et al*.^5^, that light driven signals are transmitted from the retina to visual areas of the brain in TKO mice.

In summary, our results are consistent with the persistence of residual light responses in the TKO retina that originate in rod photoreceptors, potentially a small specialised subset of rod photoreceptors that couple to a rare and distinct subtype of atypical AII amacrine cell. The functional relevance of this pathway to normal vision remains unknown, although based on the relatively low density of responsive cells it seems unlikely that this retinal circuit is capable of mediating high resolution image forming vision. Our data highlight the TKO mouse model as a valuable tool to study novel secondary light signalling pathways within the mouse retina.

## Methods

### Animals

All animal procedures were performed in accordance with the United Kingdom Animals (Scientific Procedures) Act of 1986 and the University of Oxford Policy on the Use of Animals in Scientific Research. All experiments were approved by the University of Oxford Animal Welfare and Ethical Review Board, and were conducted under PPL 30/3068. Wild type C3H mice (C3H/He; not carrying *rd1* mutation)^39^, *Opn4*^*−/−*^(*tau-LacZ*^+/+^) mice that express a β-gal reporter selectively within M1 type pRGCs^20^, degenerate *rd/rd cl* mice (>P80) lacking rod and cone photoreceptors^39^, and *Gnat*^*−/−*^, *Cnga3*^*−/−*^, *Opn4*^*−/−*^ triple knockout mice (TKO)^4^ were housed under a 12:12 LD cycle with food and water *ad libitum*.

### MEA recordings of light responses from the TKO retina

Mice were culled by cervical dislocation at ZT 6–8, followed immediately by enucleation and dissection of retinae under dim red light conditions (>610 nm) in AMES media (Sigma) bubbled with 95% O_2_ 5% CO_2_ (pH 7.4). Retina were then placed ganglion cell side down onto glass bottomed MEA chambers containing 60 electrodes each 30 μm in diameter and spaced 200 μm apart (Multi Channel Systems) and anchored in place with glass coated metal harps (ALA Scientific Instruments). MEA chambers were placed into the MEA recording device (MEA1060-Inv, Multi Channel Systems), fitted with a gas permeable perfusion manifold (ALA Scientific Instruments), and mounted onto the stage of an inverted Olympus IX71 microscope so that the recording electrodes were positioned in the microscope light path. Retinae were continuously perfused at 2 ml/minute with AMES media bubbled with 95% O_2_ 5% CO_2_ (pH 7.4) and maintained at 34 °C using a combination of water bath heater (36 °C), in-line perfusion heater (35 °C), and base plate heater incorporated into the MEA system (34 °C) to minimise temperature fluctuations in the sample chamber. Recorded signals were collected, amplified and digitized at 25 KHz using MC Rack software (Multi Channel Systems). Retinae were perfused in the dark for 60 minutes prior to recording of light responses. For all recordings baseline activity was recorded for 30–60 s prior to light stimulation. For recording of flash ERG type responses retina were stimulated with 500 ms flashes of light. For other experiments the duration of light stimuli ranged from 1–30 s (as stated). Retinae were dark adapted for 20 minutes between bouts of recordings. Monochromatic light stimuli (360 nm and 500 nm, bandwidth 20 nm) were generated by a Xenon arc light source with a slit monochromator (Cairn Optoscan) and delivered via a 10x microscope objective beneath the MEA chamber. Duration and wavelength of light stimuli were controlled via Metafluor software (Molecular Devices). Intensity of light stimuli were adjusted using neutral density filters (0 to 4 log units, Thor Labs) and controlled via an automated filter wheel (Prior Scientific) placed into the microscope light path. The power of light stimuli (μW/cm^2^/s) was measured at the sample focal plane using an in-line power meter (PM160T, Thor Labs), with power measurements converted to irradiance (photons/cm^2^/s) using an irradiance conversion toolbox (http://www.fmrib.ox.ac.uk/NLO/team/principal -investigators/stuartpeirson/downloadstile-14/filetile-3). The group III metabotropic glutamate receptor agonist L-2-amino-4-phosphonobutyric acid (L-AP4) was obtained from Tocris.

### Light-induced *c-fos* expression

Mice under normal light dark cycles were exposed to 30 minute light pulses or sham light pulses at ZT16 (4 hours into the dark phase). Following the cessation of light stimuli mice were kept in the dark for a further 30 minutes before eyes were collected and processed for immunostaining. The methods used for light pulses have been described previously^15^. Briefly, white light stimuli lacking any UV component (14.7 log photons/cm^2^/s) and 360 nm UV light stimuli (12.7 log photons/cm^2^/s) were produced by LED light sources (white light LED, BXRAC2002, Bridgelux; UV LED, NCCU033, Nichia, Japan). The spectra of each light source, and a comparison to the peak sensitivities of the mouse photopigments has been reported previously^15^. The intensity of white light and UV stimuli striking the cornea were measured using a radiometrically calibrated spectrophotometer (Ocean Optics) and an illuminance UV recorder (TR-74Ui, T&D Corporation) respectively.

### Immunostaining

Preparation and immunostaining of retina sections and whole retina flatmounts was performed as described previously^15^,^40^. Primary antibodies were incubated for 24–72 hours at 4 °C. Secondary antibodies were incubated 1:200 for 2 hours at 22 °C. All secondary antibodies were raised in donkey and conjugated with Alexa dyes (Life Technologies). A summary of primary and secondary antibodies is shown in Table 1. For retinal sections, all antibodies were diluted in PBS with 2.5% donkey serum and 0.2% Triton-X. All wash steps were performed using PBS with 0.05% Tween-20. For staining of retina flatmounts levels of Triton-X were increased to 1%. Samples were mounted in Prolong Gold anti-fade media containing DAPI (Life Technologies).

### Image acquisition

Fluorescent images were collected using a LSM 710 laser scanning confocal microscope and Zen 2009 image acquisition software (Zeiss). Individual channels were collected sequentially. Laser lines for excitation were 405 nm, 488 nm, 561 nm and 633 nm. Emissions were collected between 440–480, 505–550, 580–625 and 650–700 nm for blue, green, red and far-red fluorescence respectively. For all images, global enhancement of brightness and contrast was performed using Zen Lite 2011 image analysis software (Zeiss). For direct quantitative comparisons (where stated), all images were acquired and processed under identical conditions.

### Statistical analysis

All data are shown as mean ± SEM. Statistical analysis was performed using unpaired two-tailed Student’s t-test.

## Additional Information

**How to cite this article**: Hughes, S. *et al*. Characterisation of light responses in the retina of mice lacking principle components of rod, cone and melanopsin phototransduction signalling pathways. *Sci. Rep.* **6**, 28086; doi: 10.1038/srep28086 (2016).

## Supplementary Material

Supplementary Information

## Figures and Tables

**Table 1:** Primary and secondary antibodies.

Target	Antibody species	Antibody/Source	Dilution	Secondary antibody
Melanopsin	Rabbit polyclonal	UF006, Advanced Targeting Systems	1:2500	Donkey anti-rabbit Alexa 488
*c-fos*	Rabbit monoclonal	9F6, Cell signalling	1:200	Donkey anti-rabbit Alexa 568
*c-fos*	Sheep polyclonal	ab6167, Abcam	1:500	Donkey anti-sheep Alexa 568
*β*-gal	Chicken polyclonal	ab9361, Abcam	1:1000	Donkey anti-chicken Alexa 488
Brn3a	Goat polyclonal	sc-31985, Santa Cruz Biotech	1:1000	Donkey anti-goat Alexa 488
GS	Mouse monoclonal	MAB302, Millipore	1:1000	Donkey anti-mouse 488
Dab1	Rabbit polyclonal	LS-B9240, Lifespan Biosciences	1:1000	Donkey anti-rabbit Alexa 488
TH	Chicken polyclonal	ab76442, Abcam	1:1000	Donkey anti-chicken Alexa 488
GABA	Mouse monoclonal	GB-69, Sigma	1:2500	Donkey anti-mouse Alexa 488
GlyT-1	Goat polyclonal	AB1770, Millipore	1:1000	Donkey anti-goat Alexa 488/633
ChAT	Goat polyclonal	AB1449, Millipore	1:1000	Donkey anti-goat Alexa 488
Calbindin	Rabbit polyclonal	ab11426, Abcam	1:1000	Donkey anti-rabbit Alexa 488
CHX10	Sheep polyclonal	ab16141, Abcam	1:500	Donkey anti-sheep Alexa 488

Details of primary antibodies used for immunohistochemistry. *β*-gal; beta-galactosidase, Brn3a; Brain-specific homeobox POU domain protein 3A, GS; Glutamine synthetase, Dab1; Disabled-1, TH; Tyrosine hydroxylase, GABA; Gamma-Aminobutyric acid, GlyT-1; Glycine Transporter-1, ChAT; Choline acetyltransferase, CHX10; Homeodomain transcription factor ChX10. Note that two different *c-fos* antibodies raised in different species were employed to allow double labelling with different combinations of primary antibodies.
