# Supplementary material for: Characterisation of light responses in the retina of mice lacking principle components of rod, cone and melanopsin phototransduction signalling pathways
Source: Sci Rep. 2016 Jun 15;6:28086. doi: 10.1038/srep28086 (PMC4908426; doi:10.1038/srep28086)
Supplement: Supplementary Information [file srep28086-s1.pdf]

Characterisation of light responses in the retina of mice lacking principle components of rod, cone and melanopsin phototransduction signalling pathways.

Steven Hughes, Jessica Rodgers, Doron Hickey, Russell G. Foster, Stuart N. Peirson and Mark W. Hankins

The Nuffield Laboratory of Ophthalmology, Sleep and Circadian Neuroscience Institute, Nuffield Department of Clinical Neurosciences, University of Oxford, Sir William Dunn School of Pathology, OMPI G, South Parks Road, Oxford, OX1 3RE, UK

Correspondence to mark.hankins@eye.ox.ac.uk

Supplementary Material

Supplementary Figure 1

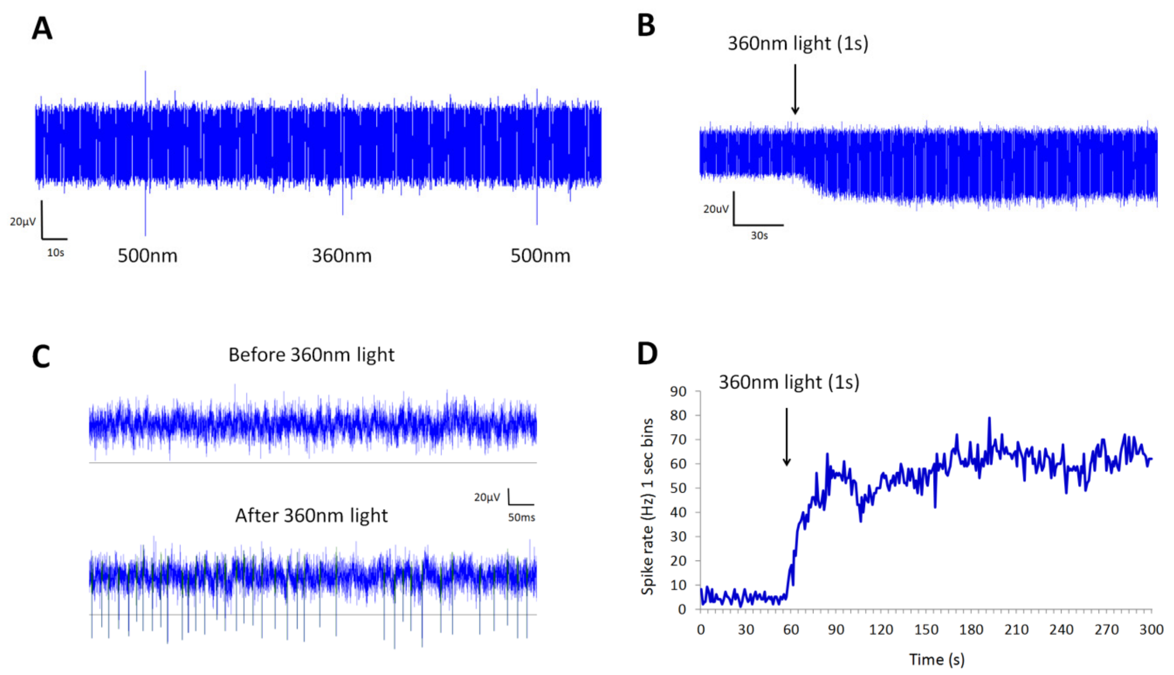

Supplementary Figure 1. MEA recordings from the TKO retina following stimulation with UV light

A) Raw electrode data showing micro ERG type responses elicited by sequential pulses of 500nm, 360nm and 500nm light 60 s apart (500ms, 15.1 log photons/cm^2/s and 14.9 log photons/cm^2/s light for 500nm and 360nm stimuli respectively). **B)** A single electrode (1 of 222 electrodes from n=4 retina) showed a marked increase in action potential spike firing following exposure to UV light (1 sec, 360nm, 14.9 log photons/cm^2/s). **C)** Examples of action potential spikes detected before and after light exposure, from trace shown in B. **D)** Graph showing the change in spike firing rate over time (1 s bins) detected from the electrode shown in B.

Supplementary Figure 2

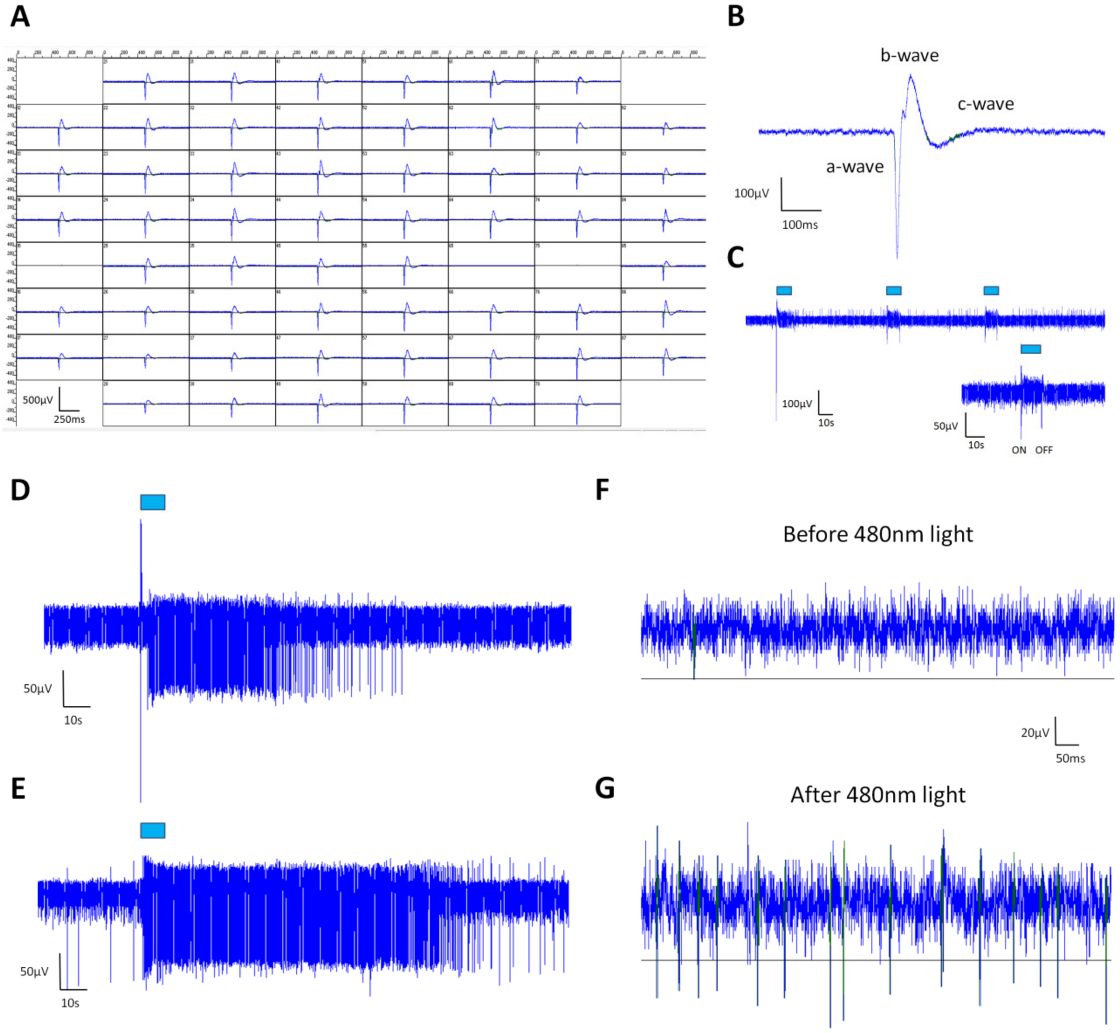

Supplementary Figure 2. MEA recordings from wild type and degenerate *rd/rd cl* retina.

A) Image showing micro ERG type responses recorded from wild type retina following stimulation with 500nm light (500ms, 15.1 log photons/cm^2/s). Panels show the responses recorded from all recording electrodes of a single MEA chamber. Micro ERG events were larger in amplitude compared to those recorded from TKO retina, and were typically observed on all individual electrodes. **B**) An example of a micro ERG response recorded from the wild type retina showing the principle components of the ERG response (500nm, 500ms, 15.1 log photons/cm^2/s). **C**) Raw electrode data showing responses elicited from wild type retina by multiple 500nm light pulses (10s, 15.1 log photons/cm^2/s). Note the rapid bleaching of the rod driven *a*-wave, and the presence of cone driven ON and OFF responses that are smaller and typically show limited bleaching. **D**) Raw electrode data showing both a micro ERG type response and a melanopsin type response recorded from the wild type retina (500nm, 10s, 15.1 log photons/cm^2/s). **E)** Raw electrode data showing melanopsin type responses recorded from the degenerate *rd/rd cl* retina (480nm, 10s, 15.1 log photons/cm^2/s). Note the lack of ERG type response at light onset. **F-G**) Examples of action potential spikes detected in the *rd/rd cl* retina before and after 480nm light exposure, from trace shown in E.

Supplementary Figure 3

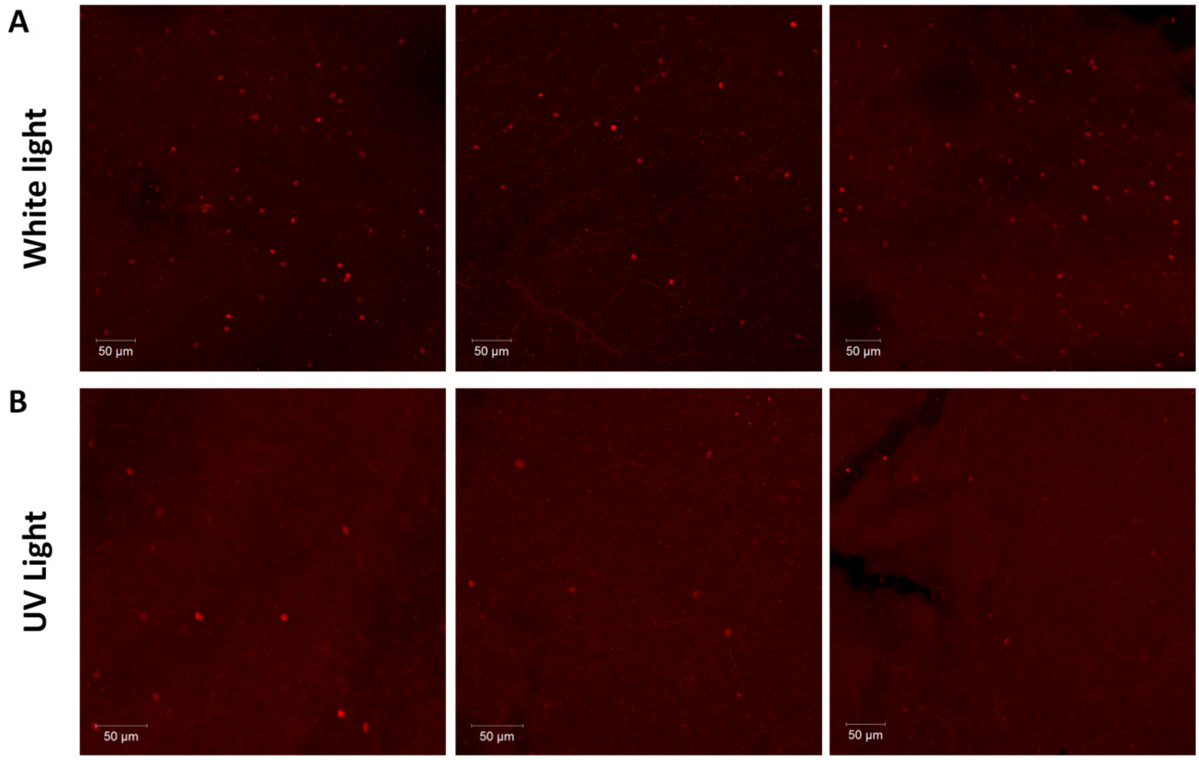

Supplementary Figure 3. Light-induced *c-fos* expression in the TKO retina following white light and UV light pulses.

A-B) Images showing levels of light-induced *c-fos* expression detected in the TKO retina following white light (14.9 log photons/cm^2/s, 30 min at ZT 16) (**A**) and UV light stimuli (12.7 log photons/cm^2/s, 30 min at ZT 16). (**B**). Images shown are collected from n=3 replicate retina. Flatmount images are generated from merging confocal slices (1µm) collected from the ganglion cell layer to the inner nuclear layer.

Supplementary Figure 4

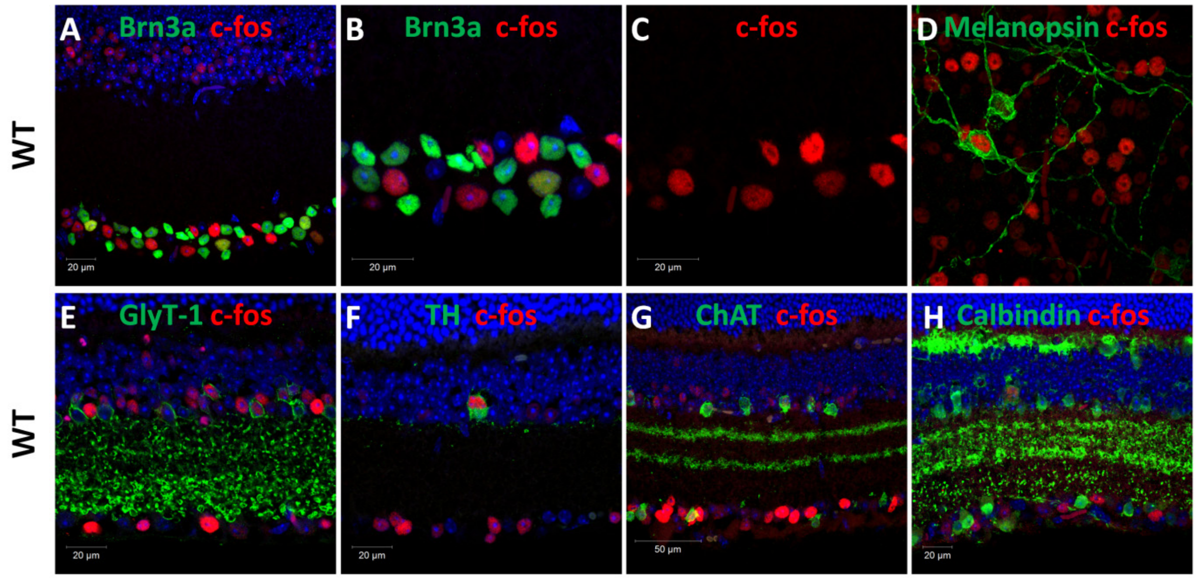

Supplementary Figure 4. Multiple cell types show light-induced expression of *c-fos* in the wild type retina

A-C) *c-fos* expression in Brain-specific homeobox POU domain protein 3A (Brn3a) positive retinal ganglion cells. **D)** *c-fos* expression in M1-type melanopsin expressing pRGCs (identified by expression of β-galactosidase reporter in the TKO retina). **E)** *c-fos* expression in glycine transporter-1 (GlyT-1) positive glycinergic amacrine cells. **F**) *c-fos* expression in tyrosine hydroxylase (TH) positive dopaminergic amacrine cells. **G**) *c-fos* expression in choline acetyltransferase (ChAT) positive starburst amacrine cells. **H**) *c-fos* expression in calbindin positive amacrine cells. Overall the highest levels of *c-fos* expression are typically observed for amacrine cells located within the GCL, and TH positive dopaminergic amacrine cells located in the INL. Lower levels of *c-fos* are observed for Brn3a positive RGCs. DAPI nuclear counterstain is shown in blue. For all panels, results are shown following stimulation with white light (14.9 log photons/cm^2/s, 30 min at ZT 16).

Supplementary Figure 5

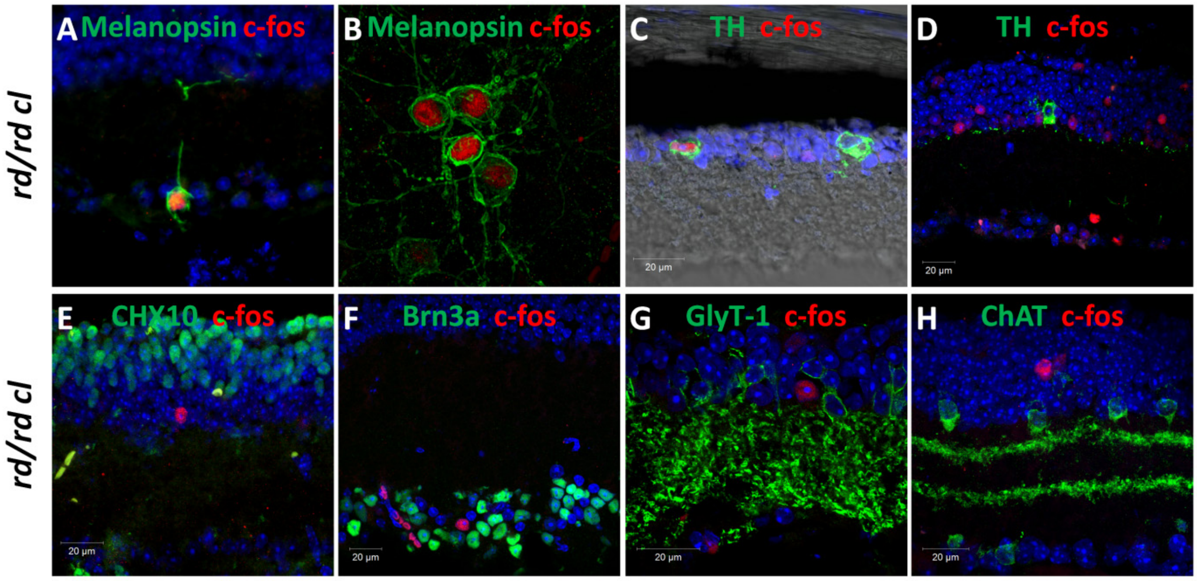

Supplementary Figure 5. Light-induced expression of *c-fos* in the degenerate *rd/rd cl* retina is largely restricted to pRGCs

A-B) Light-induced *c-fos* expression in the degenerate *rd/rd cl* retina is largely restricted to melanopsin expressing pRGCs (typically located in the GCL). **C-D)** A number of other cell types are *c-fos* positive in the *rd/rd cl* retina which receive retrograde signals from pRGCs, including a small proportion of tyrosine hydroxylase (TH) positive dopaminergic amacrine cells (~5-10%) but not CHX10 positive bipolar cells (**E**) Brain-specific homeobox POU domain protein 3A (Brn3a) positive retinal ganglion cells (**F**) glycine transporter-1 (GlyT-1) positive amacrine cells (**G**) or choline acetyltransferase (ChAT) positive starburst amacrine cells (**H**). DAPI nuclear counterstain is shown in blue. For all panels, results are shown following stimulation with white light (14.9 log photons/cm^2/s, 30 min at ZT 16).
